# A ^99m^Tc-labelled scFv antibody fragment that binds to prostate-specific membrane antigen

**DOI:** 10.1097/MNM.0000000000000698

**Published:** 2017-05-31

**Authors:** Saima Nawaz, Gregory E.D. Mullen, Philip J. Blower, James R. Ballinger

**Affiliations:** a Division of Imaging Sciences and Biomedical Engineering, King’s College London; b Department of Nuclear Medicine, Guy’s and St Thomas’ Hospital, London, UK

**Keywords:** J591, molecular imaging, PC3LN3, prostate carcinoma, prostate-specific membrane antigen, single-chain variable fragment, technetium-99m

## Abstract

**Introduction:**

Prostate-specific membrane antigen (PSMA) is an extensively studied antigen for imaging prostate cancer. We prepared a single-chain variable fragment (scFv) of J591, a monoclonal antibody that recognises an external epitope of PSMA, incorporating a His-tag for labelling with ^99m^Tc tricarbonyl, and evaluated its binding using human PCa cell lines.

**Methods:**

J591(scFv) was expressed in HEK-293T cells and purified by metal ion affinity chromatography, followed by size exclusion chromatography. Stability and monomer/dimer ratios of purified scFv under different storage conditions were analysed by SDS-PAGE and analytical size exclusion chromatography. J591(scFv) was labelled with  at 37°C for 60 min. The stability of ^99m^Tc-scFv in human serum was analysed by SDS-PAGE with autoradiography. Cell-binding studies were carried out using PC3LN3 (PSMA negative) and PC3LN3-PSMA (a variant engineered to express PSMA) cell lines.

**Results:**

J591(scFv) was most stable to dimerisation on storage at −80°C compared with −20 and 4°C. Radiochemical yields of 85–90% were obtained with the final radiochemical purity of more than 99% after purification by gel filtration. In these small-scale studies, the maximum specific activity achieved was 7 MBq/μg. Liquid chromatography–mass spectrometry showed the formation of ^99m^Tc-J591(scFv), which was radiochemically stable in serum, with no dissociation of ^99m^Tc over 24 h. Cell-binding assays showed specific binding to PSMA-positive cells.

**Conclusion:**

J591(scFv) can be radiolabelled with  conveniently and efficiently. The labelled product was stable in serum. It showed selective binding to PSMA-positive cells compared with PSMA-negative cells. This potential radiotracer warrants evaluation in PCa xenograft models.

## Abstract 2

Supplemental Digital Content is available in the text.

## Introduction

The most extensively studied antigen in prostate cancer (PCa) for antibody-based imaging is prostate-specific membrane antigen (PSMA) 1. Radiolabelled monoclonal antibodies (mAb) against PSMA have been used in the clinic for the evaluation of PCa, primarily the ^111^In-labelled monoclonal antibody, capromab pendetide (Prostascint), which has been commercially available since 1997 for SPECT imaging 2. Although the hybrid technology of SPECT/CT has improved the accuracy of ^111^In-capromab imaging, capromab has the limitation that it binds to an internal epitope of PSMA 3. J591 is an alternative antibody that recognises an extracellular domain of PSMA and has been investigated for localisation and staging of PCa 4,5. Indeed, there have been recent reports of clinical trials with ^111^In-DOTA-J591 for SPECT imaging, ^89^Zr-DFO-J591 for PET imaging and ^177^Lu-DOTA-J591 for therapy 6–8.

However, the biokinetics of mAb such as J591 are not compatible with the use of short-lived radiolabels. For example, optimal images with ^111^In-J591 were obtained 5–7 days after injection 6. To improve upon the slow kinetics of mAb, small molecule ligands for PSMA have been investigated in recent years. Molecular Insight Pharmaceuticals (now part of Progenics) have developed a range of ^99m^Tc and ^123^I-labelled PSMA ligands for imaging and ^131^I-labelled analogues as potential targeted therapeutic agents 9–11. The first human results with ^123^I MIP-1072 and MIP-1095 show rapid accumulation and prolonged retention in PSMA-expressing lesions, with tumour/background ratios of ∼10 achieved in SPECT images obtained 4 h after injection 9. In parallel work, ^68^Ga-labelled urea-based PSMA inhibitors have shown great promise for PSMA-specific tumour imaging with PET 11,12 and analogues labelled with ^177^Lu are being evaluated for therapy 13. Importantly, given the 68-min half-life of ^68^Ga, diagnostic images can be obtained within 60 min after injection 12.

The engineering of single-chain variable region fragments (scFv, *M*_Wt_: ∼27 kDa) of mAb is another route to improve their kinetics for imaging with radionuclides with short half-lives 14. Parker *et al.* 15 described the construction of a J591(scFv) based on the reported complementarity determining region of J591. The nucleotide sequence was optimised for expression in *Pichia pastoris* and a hexa His-tag was added to facilitate affinity purification. J591(scFv) has also been used as a targeting agent for the delivery of toxins to PCa 16,17. Antibody fragments such as scFv can be conveniently labelled with ^99m^Tc tricarbonyl through a His-tag. The basis of ^99m^Tc tricarbonyl kit formulation is disodium boron carbonate, which serves as a CO source and a reducing agent 18. The group at the Paul Scherrer Institute developed a direct labelling protocol for scFv and minibodies carrying an N-terminal or a C-terminal His-tag with ^99m^Tc tricarbonyl 19 and further optimisation has been performed by Badar *et al.* 20.

In an editorial on agents for imaging PCa, Eder *et al.* 11 stated that PSMA is the most promising target because of its accessibility on the cell surface, internalisation following binding for retention of the radiolabel and correlation of expression with stage and grade of PCa. With this objective, we have developed a ^99m^Tc-labelled scFv of the mAb J591 and evaluated its binding to PSMA-expressing human PCa cells.

## Materials and methods

### Protein production and purification

The J591(scFv) sequence in the VH-VL orientation was PCR amplified from the SFG P28z vector as described previously 21. Mammalian HEK-293T cells were used to produce J591(scFv) in a growth medium of RPMI 1640 (PAA) with 10% FBS and antibiotics. The cells were cultured in an atmosphere of 5% CO_2_ at 37°C. The supernatant was collected over 4–6 weeks and stored at 4°C. When ∼1500 ml of supernatant had been collected, it was purified by Ni-NTA chromatography (Qiagen, Manchester, UK), followed by size exclusion FPLC (AKTA, Superdex 75 10/300 GL; GE Life Sciences, Little Chalfont, UK). The elution fractions were concentrated using centrifugal concentrators (Vivaspin 6 PES, MWCO 5000; Fisher-Sartorius, Loughborough, UK) before exchanging the buffer to PBS (pH 7) by size exclusion chromatography (as above). The protein was analysed by SDS-PAGE (Novex, Fisher, Loughborough, UK) using 12% gel, western blotting with 3,3-diaminobenzidine peroxidase (Sigma-Aldrich, Gillingham, UK) to detect the HRP-conjugated secondary antibody and HPLC (SEC-2000; Phenomenex, Macclesfield, UK). The protein was stored at −80°C in small aliquots on the basis of the protein concentration, which was determined using a UV–visible Spectrophotometer Nanodrop 2000c (Thermo Fisher Scientific, Paisley, UK). For stability studies, aliquots were also stored at 4°C and −20°C for 3 weeks. The effect of addition of 5% glycerol to reduce the extent of dimerisation was also evaluated.

### Radiochemistry

^99m^Tc tricarbonyl was prepared by adding 1000 MBq ^99m^Tc-pertechnetate (Drytec generator; GE Healthcare, Little Chalfont, UK) in 500 µl to an Isolink kit (donated by Covidien Healthcare, Fareham, UK)) and heating it for 25 min at 100°C. The formation of the ^99m^Tc tricarbonyl was monitored by TLC on glass-backed silica gel 60 plates developed with 1% HCl in methanol 20. The *R*_f_ values in this system are as follows: ^99m^Tc tricarbonyl, 0.2–0.8; ^99m^Tc colloids, 0.0 and ^99m^Tc-pertechnetate, 0.9. An aliquot of the ^99m^Tc tricarbonyl (∼200 MBq in 100 µl) was then incubated with 100 µl J591(scFv) (1 µg/µl) for 60 min at 37°C. After radiolabelling, the reaction mixture was applied to a size exclusion column (Sephadex G-25 PD Mini-trap; GE Life Sciences, Little Chalfont, UK), eluted with PBS and 30 fractions of 100 µl each were collected by gravity as described previously 22. The activity in the fractions was measured in an ionisation chamber (CRC-15R; Capintec, Southern Scientific, Henfield, UK). The fractions with the highest activity (generally 8, 9 and 10) were analysed on iTLC-SA strips (Agilent Technologies, Cheadle, UK) developed with 0.1 mol/l citrate buffer pH 5.5 and analysed by a radiochromatogram scanner (BioScan, Washington DC, USA). The *R*_f_ values in this system are as follows: ^99m^Tc-J591(scFv), 0.0; ^99m^Tc tricarbonyl, 0.9 and ^99m^Tc-pertechnetate, 0.9. The fractions with the highest activity were then pooled.

A series of experiments were conducted that involved radiolabelling of J591(scFv) (10 µg) with different amounts of ^99m^Tc tricarbonyl (10, 30, 50 and 70 MBq) to determine the maximum amount of radioactivity that could be loaded onto the J591(scFv). In another series, different concentrations of J591(scFv) ranging from 72 to 3.6 µmol/l were reacted with the same amount of tricarbonyl (10 µl, 30 MBq). Finally, J591(scFv) was radiolabelled with ^99m^Tc tricarbonyl in two different NaCl concentrations (140 and 500 mmol/l) to determine the effect of salt concentration on radiolabelling efficiency 20.

J591(scFv) and ^99m^Tc-J591(scFv) were characterised by mass spectrometry (MS) (Series 6520 QTOF LC–MS; Agilent Technologies, Cheadle, UK). The unlabelled scFv (20 µl, ∼30 µg) was analysed by direct injection and ^99m^Tc-J591(scFv) (1 MBq, 10 µl, ∼16 µg) was analysed by liquid chromatography–mass spectrometry on a C18 column with an acetonitrile/0.1% formic acid gradient from 0 to 100% acetonitrile over 15 min at a flow rate of 0.5 ml/min.

Serum stability was studied by incubating ^99m^Tc-J591(scFv) with an equal volume of human serum at 37°C and removing aliquots over the course of 24 h. Samples were tested for release of free pertechnetate or other small molecules containing ^99m^Tc by iTLC-SA as described above; however, this method would not detect transchelation to serum proteins as both ^99m^Tc-J591(scFv) and serum proteins would remain at the origin. Samples were analysed by SDS-PAGE 12% gel with detection by a phosphorimager (Cyclone Plus; Perkin Elmer, Beaconsfield, UK) and Coomassie blue staining.

### Cell-binding studies

The PC3LN3 (parental, PSMA negative) and PC3LN3-PSMA (a variant engineered to express PSMA) cell lines were produced as described previously 22. FACS Calibur (Becton Dickinson, Oxford, UK) analysis before the cell-binding experiments with PCLN3 and PC3LN3-PSMA cell lines confirmed PMSA status using the anti-His-tag antibody penta-His Alexa 488 (Qiagen). For cell-binding experiments, 24-well plates were seeded with equal numbers of cells (4×10^5^) in a volume of 500 µl/well and maintained at 37°C for 24 h. To achieve a range of protein concentrations, 10 serial dilutions of ^99m^Tc-J591(scFv) were prepared in triplicate from 7200 nmol/l (10 µg in 50 µl) to 0.4 nmol/l (0.001 µg in 50 µl). The cells were incubated with ^99m^Tc-J591(scFv) for 60 min at 4°C to minimise internalisation, after which the supernatant was aspirated and the cells were carefully washed three times with 500 µl Hanks’ balanced salt solution to remove any unbound radioactivity. The cells were then lysed with 0.5 mol/l NaOH (200 µl) and the lysate was transferred to tubes for gamma counting (1470 Wallac Wizard; Perkin Elmer). The percentage bound was plotted against protein concentration and the results were analysed by nonlinear regression using GraphPad Prism software (San Diego, Califonia, USA) with a one-site total binding algorithm to calculate an EC_50_ value for the cold mAb.

### Statistical analysis

Results are expressed as mean±SD for *n* independent experiments. Differences between groups were assessed using Student’s *t*-test for unpaired values.

## Results

### Protein production and purification

J591(scFv) was produced in mammalian cells. A yield of 10–12 mg/l of J591(scFv) was obtained from the supernatant after the purification steps of the Ni-NTA column and SEC. In the Ni-NTA purification step, J591(scFv) eluted as a single peak when the column was washed with 250 mmol/l imidazole. In the SEC buffer adjustment step, the dimer fraction eluted first (10–15 ml), followed by the monomer fraction (15–20 ml) (Supplementary Fig. S1a, Supplemental digital content 1, *http://links.lww.com/NMC/A116*). An intense band observed at 27.7 kDa in both SDS-PAGE (Supplementary Fig. S1b, Supplemental digital content 1, *http://links.lww.com/NMC/A116*) and western blotting (Nawaz S, Kampmeier F, Mullen GED, Blower PJ and Ballinger JR, unpublished data) also confirmed the results. The purified sample was further analysed by size exclusion HPLC. The monomer fraction eluted at 9 min and the dimer at 8 min The problem of dimerisation over a period of 3 weeks was minimised by immediate storage at −80°C compared with 4°C and −20°C (Nawaz S, Kampmeier F, Mullen GED, Blower PJ and Ballinger JR, unpublished data). The addition of glycerol did not significantly decrease the dimerisation of J591(scFv).

### Radiochemistry

For the preparation of ^99m^Tc tricarbonyl, pertechnetate was added to the kit and heated for 25 min at 100°C. TLC showed an 85–90% radiochemical yield of ^99m^Tc tricarbonyl, with the main impurity being free pertechnetate. The product was used for the subsequent labelling reaction without purification.

**Fig. 1:**
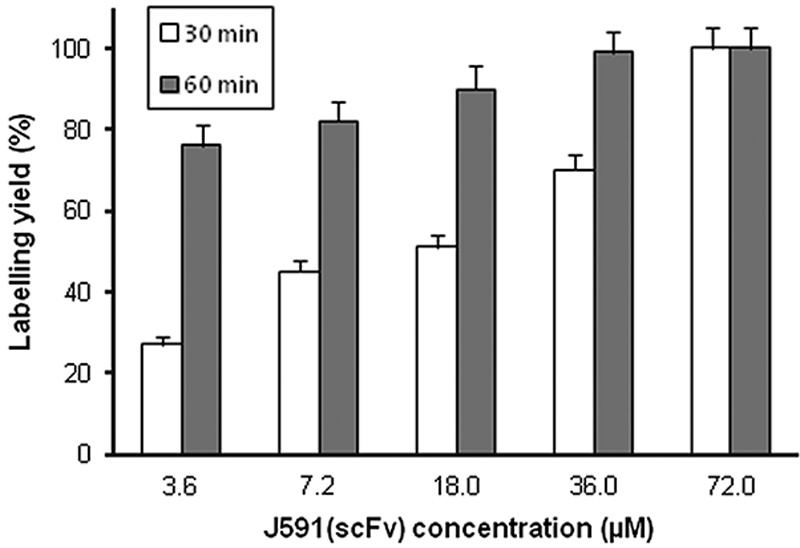
Effect of J591(scFv) concentration on labelling efficiency after 30 and 60 min of incubation. Each value is mean+SD for three measurements.

**Fig. 2:**
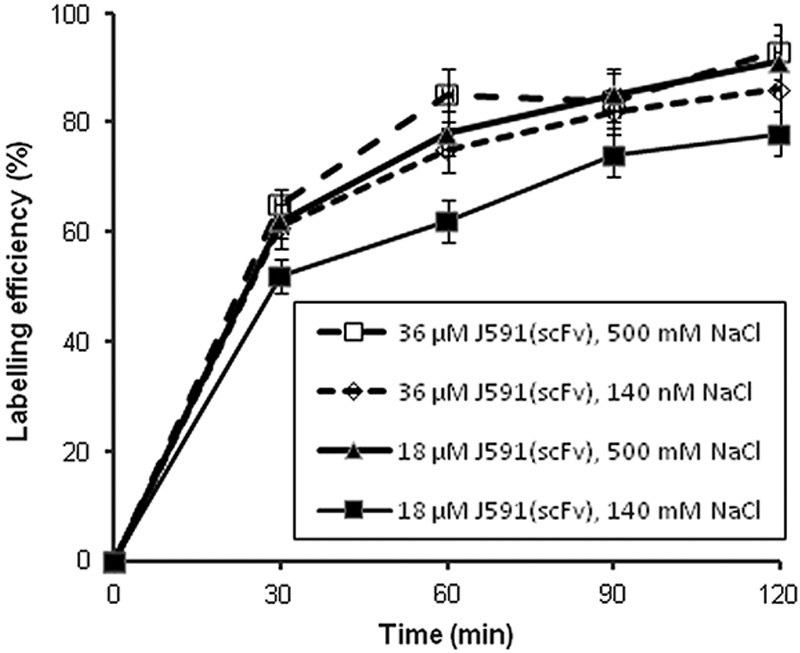
Effect of NaCl concentration (140 or 500 mmol/l) on the labelling efficiency of J591(scFv) (18 or 36 µmol/l) with ^99m^Tc tricarbonyl. Each data point is mean±SD for three measurements.

Site-specific labelling with ^99m^Tc tricarbonyl via the His-tag of J591(scFv) resulted in a radiochemical yield of 85–100%. If the yield was less than quantitative, a final radiochemical purity of more than 99% could be achieved after a gel filtration purification step (Supplementary Fig. S2, Supplemental digital content 1, *http://links.lww.com/NMC/A116*). The radiochemical yield increased during incubation at 37°C up to 60 min, after which there was no further increase. For higher concentrations of J591(scFv), the time of incubation had no effect on the radiolabelling yield. However, with low concentrations, the duration of incubation played an important role (Fig. 1). For example, with 3.6 µmol/l J591(scFv), the radiochemical yield increased from 27±2% at 30 min to 80±5% at 60 min (*n*=3, *t*-test, *P*=0.005), whereas at 72 µmol/l, radiochemical yield was ∼100% at both time points (*P*=NS). The maximum amount of ^99m^Tc tricarbonyl that could be loaded onto 1 µg J591(scFv) was 7 MBq. As previous work from this laboratory showed that the NaCl concentration can influence labelling efficiency with ^99m^Tc tricarbonyl 20, experiments were conducted at two different salt concentrations. Increasing the NaCl concentration from 140 to 500 mmol/l slightly, but not significantly, increased the radiolabelling yield (Fig. 2). With 36 µmol/l J591(scFv), the radiochemical yield after 60 min of incubation was 75±4% at 140 mmol/l NaCl and 85±5% at 500 mmol/l NaCl (*n*=3, *t*-test, *P*=NS). ^99m^Tc-J591(scFv) was radiochemically stable in serum, with no change in the speciation of ^99m^Tc over 24 h as shown by TLC and SDS-PAGE, where there remained only an intense band at ∼30 kDa.

Analysis by MS following a direct injection of J591(scFv) showed a strong peak at 27.7 kDa, which corresponds to the molecular weight of the J591(scFv). Liquid chromatography–mass spectrometry, using a C18 column, showed the formation of ^99m^Tc-J591(scFv) detected in the radiometric chromatogram. MS of ^99m^Tc-J591(scFv) showed a single peak at 27.7 kDa.

### Cell-binding studies

**Fig. 3:**
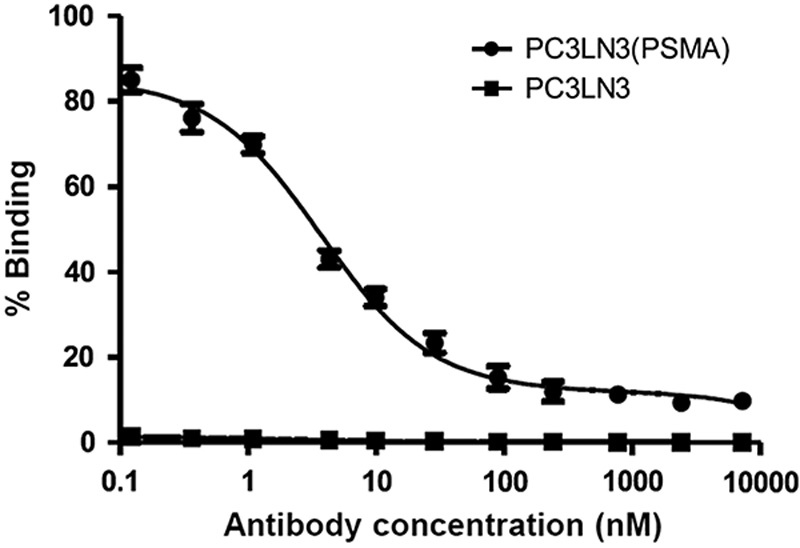
Binding of ^99m^Tc-J591(scFv) to PC3LN3 parental and PC3LN3-PSMA overexpressing cell lines. Each data point is mean±SD for three measurements.

Before conducting the cell-binding experiments, both the negative and the PSMA-positive cell lines were analysed by FACS for PSMA expression. The PC3LN3-PSMA cell line showed a positive shift in FL1, whereas the parental PSMA-negative PC3LN3 showed no shift in FL1. Serial dilutions of ^99m^Tc-J591(scFv) were incubated with the both cell lines at 4°C, to minimise internalisation, for 60 min for binding equilibrium to be reached. As can be seen in Fig. 3, the binding of ^99m^Tc-J591(scFv) showed that J591(scFv) specifically and saturably binds to the PC3LN3-PSMA cell line with an EC_50_ value of 3.7 nmol/l, whereas only nonspecific binding was observed in the PC3LN3 cell line.

## Discussion

The role of molecular imaging in localisation and staging of PCa is becoming increasingly important, with a variety of probes under development 11. Although recent interest has focused on small molecules, there may be a role for antibody-based targeting and the preparation of a radiolabelled scFv fragment may offer a suitable balance between binding affinity and pharmacokinetics suited to short half-lives such as those of ^99m^Tc or ^68^Ga.

J591(scFv) was conveniently produced in mammalian cells at a moderate yield of 10–12 mg/l of J591(scFv). Purification by an Ni-NTA column, followed by size exclusion chromatography yielded the monomer, which was characterised by SDS-PAGE, western blotting and SEC. The product could be stored at −80°C for at least 3 weeks with minimal dimerisation of J591(scFv).

Site-specific labelling of the His-tag of J591(scFv) was performed by a two-step procedure. First, ^99m^Tc tricarbonyl was prepared in a good yield by the addition of ^99m^Tc-pertechnetate to a kit and heating. The ^99m^Tc tricarbonyl was then incubated with J591(scFv), resulting in a radiochemical yield of 85–100%. At low protein concentrations, the radiochemical yield increased during incubation at 37°C up to 60 min, after which there was no further increase. For high concentrations of J591(scFv), the time of incubation had no effect on the radiolabelling yield. A high NaCl concentration (500 mmol/l) modestly increased the radiolabelling yield as we have reported previously 20.

A final radiochemical purity of more than 99% could be achieved after a gel filtration purification step in which a Sephadex G-25 column removed any free pertechnetate or tricarbonyl from the reaction mixture. This was particularly important for the serum stability experiments, although there was some loss of radiolabelled compound on the column (recovery ∼80%). J591(scFv) was radiochemically stable in serum, with no change in the speciation of ^99m^Tc over 24 h.

Cell-binding experiments were carried out using negative and PSMA-positive PCa cell lines. In addition to specific binding to the receptor, nonspecific binding can also occur because of hydrophobic and ionic interactions with other sites on the cell surface, and it is important to identify the contribution of specific and nonspecific binding towards the total binding observed. Nonspecific binding was measured using the negative cell line. Measurement of the binding of J591(scFv) using ^99m^Tc-J591(scFv) showed specific, saturable binding to the PC3LN3-PSMA cell line with an EC_50_ value of 3.7 nmol/l, whereas only nonspecific binding was observed in the PC3LN3 cell line (Fig. 3). Although this does not provide an EC_50_ or an affinity value for ^99m^Tc-J591(scFv) itself, it shows that ^99m^Tc-J591(scFv) binds specifically to PC3LN3-PSMA cells in a qualitatively similar manner to J591(scFv).

In parallel with this work, we have been evaluating the potential utility of a diabody (*M*_Wt_: 54 kDa) generated from J591 22. It was labelled with ^99m^Tc tricarbonyl in a manner similar to the present work and at a similar specific activity. Specific binding of the diabody to a PSMA overexpressing variant of the DU145 cell line was found, with an IC_50_ of 5 nmol/l, very similar to the value observed in the present work. The ^99m^Tc-labelled diabody was evaluated in SCID beige mice bearing DU145-PMSA xenografts with SPECT imaging, followed by biodistribution studies. Blood pool clearance was somewhat slow and tumours were not visualised until 4 h after injection, with optimal contrast at 8 h, at which time the tumour/blood ratio was 8 and the tumour/muscle ratio was 17 22. This indicates the feasibility of the approach, but suggests that the scFv described in the present work might be more suitable as scFv fragments typically show more rapid blood clearance than a diabody.

## Conclusion

J591(scFv) can be radiolabelled with ^99m^Tc tricarbonyl conveniently and efficiently. In these small-scale studies, the maximum specific activity achieved was 7 MBq/μg. The labelled product was stable in serum. It showed selective, saturable binding to PSMA-positive cells compared with PSMA-negative cells. This potential radiotracer warrants *in-vivo* evaluation in PCa xenograft models.

## Supplementary Material

SUPPLEMENTARY MATERIAL

Supplemental digital content is available for this article. Direct URL citations appear in the printed text and are provided in the HTML and PDF versions of this article on the journal's website (*www.nuclearmedicinecomm.com*).
