# Supplementary material for: A 99mTc-labelled scFv antibody fragment that binds to prostate-specific membrane antigen
Source: Nucl Med Commun. 2017 May 31;38(8):666–71. doi: 10.1097/MNM.0000000000000698 (PMC5508854; doi:10.1097/MNM.0000000000000698)
Supplement: SUPPLEMENTARY MATERIAL [file mnm-38-666-s001.pdf]

Supplementary Figures for:

A ^{99m}Tc labelled scFv antibody fragment that binds to prostate specific membrane antigen

Short title: ^{99m}Tc labelled scFv that binds to prostate specific membrane antigen

Saima Nawaz,[a] Florian Kampmeier,[a] Gregory E D Mullen,[a] Philip J Blower,[a] and James R Ballinger[b]

[a]Division of Imaging Sciences and Biomedical Engineering, King's College London, London, UK; and [b]Department of Nuclear Medicine, Guy's and St Thomas' Hospital, London, UK

Corresponding author:
Professor P J Blower, Division of Imaging Sciences and Biomedical Engineering, King's College London, 4th floor Lambeth Wing, St Thomas Hospital, Westminster Bridge Road, London, UK, SE1 7EH. Tel +44 207 188 9513, Fax +44 207 188 5442; E-mail philip.blower@kcl.ac.uk

Supplementary Figure S1A

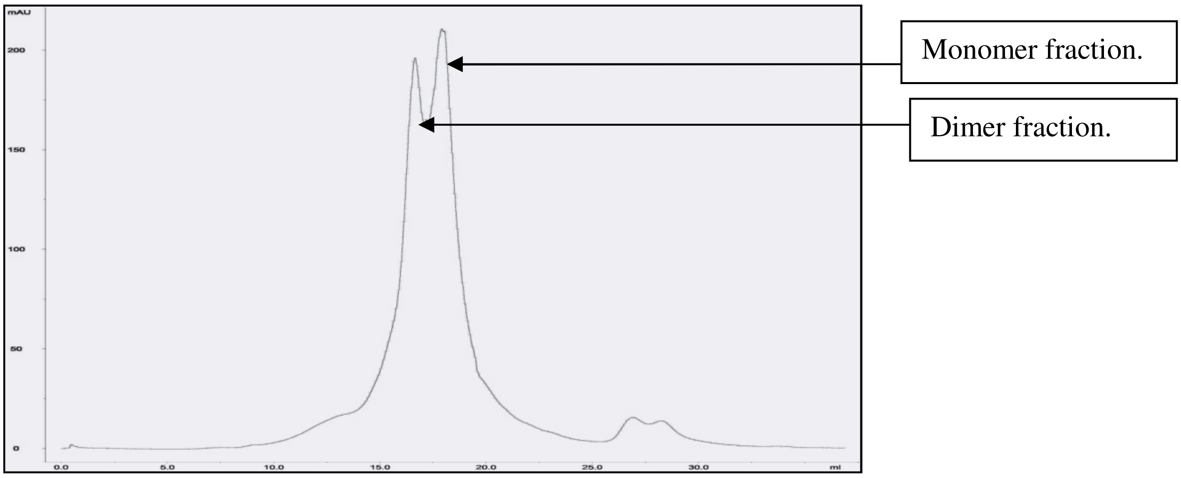

Supplmentary Figure S1B

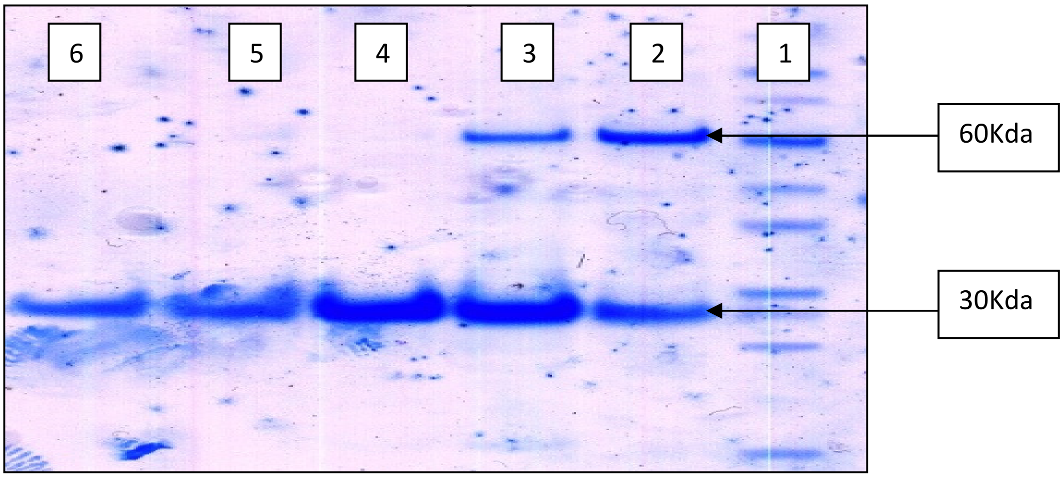

Supplementary Figure S1. A) Size-exclusion FPLC chromatogram showing elution of dimer and monomer forms of J591(scFv). B) SDS-PAGE analysis of FPLC fractions: 1, molecular weight markers; 2, fraction 8 (monomer and dimer); 3, fraction 9 (monomer and dimer); 4, fraction 10 (monomer); 5, fraction 11 (monomer); 6, fraction 12 (monomer).

Supplementary Figure S2

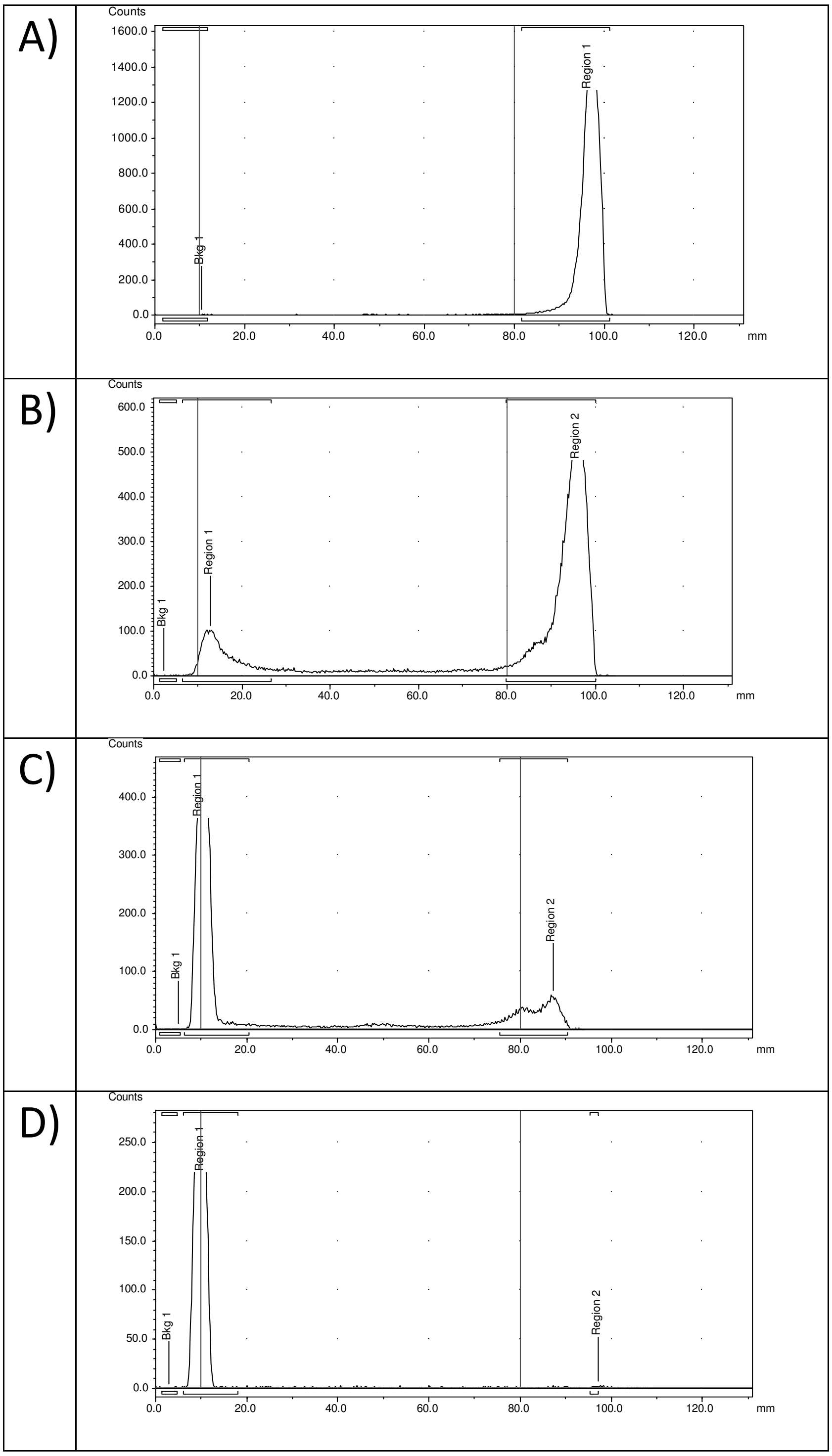

Supplementary Figure S2. Radiochromatograms on ITLC-SA strips developed with citrate buffer: A) ^{99m}Tc pertechnetate; B) ^{99m}Tc tricarbonyl; C) ^{99m}Tc-J591(scFv) reaction mixture; D) ^{99m}Tc-J591(scFv) following gel filtration column purification.
